# Oral Ondansetron Administration in Emergency Departments to Children with Gastroenteritis: An Economic Analysis

**DOI:** 10.1371/journal.pmed.1000350

**Published:** 2010-10-12

**Authors:** Stephen B. Freedman, Michael J. Steiner, Kevin J. Chan

**Affiliations:** 1 Division of Pediatric Emergency Medicine, The Hospital for Sick Children, Toronto, Ontario, Canada; 2 Division of Gastroenterology, Hepatology and Nutrition, The Hospital for Sick Children, Toronto, Ontario, Canada; 3 The University of Toronto, Toronto, Ontario, Canada; 4 Department of Pediatrics, The University of North Carolina School of Medicine, Chapel Hill, North Carolina, United States of America; 5 Munk Centre for Global Studies, Toronto, Ontario, Canada; Aga Khan University, Pakistan

## Abstract

**Background:**

The use of antiemetics for children with vomiting is one of the most controversial decisions in the treatment of gastroenteritis in developed countries. Ondansetron, a selective serotonin receptor antagonist, has been found to be effective in improving the success of oral rehydration therapy. However, North American and European clinical practice guidelines continue to recommend against its use, stating that evidence of cost savings would be required to support ondansetron administration. Thus, an economic analysis of the emergency department administration of ondansetron was conducted. The primary objective was to conduct a cost analysis of the routine administration of ondansetron in both the United States and Canada.

**Methods and Findings:**

A cost analysis evaluated oral ondansetron administration to children presenting to emergency departments with vomiting and dehydration secondary to gastroenteritis from a societal and health care payer's perspective in both the US and Canada. A decision tree was developed that incorporated the frequency of vomiting, intravenous insertion, hospitalization, and emergency department revisits. Estimates of the monetary costs associated with ondansetron use, intravenous rehydration, and hospitalization were derived from administrative databases or emergency department use. The economic burden in children administered ondansetron plus oral rehydration therapy was compared to those not administered ondansetron employing deterministic and probabilistic simulations. We estimated the costs or savings to society and health care payers associated with the routine administration of ondansetron. Sensitivity analyses considered variations in costs, treatment effects, and exchange rates. In the US the administration of ondansetron to eligible children would prevent approximately 29,246 intravenous insertions and 7,220 hospitalizations annually. At the current average wholesale price, its routine administration to eligible children would annually save society US$65.6 million (US$49.1–US$81.1) and health care payers US$61.1 million (US$46.2–US$76.3). In Canada the administration of ondansetron to eligible children would prevent 4,065 intravenous insertions and 1,003 hospitalizations annually. Its routine administration would annually save society CDN$1.72 million (CDN$1.15–CDN$1.89) and the health care system CDN$1.18 million (CDN$0.88–CDN$1.41).

**Conclusions:**

In countries where intravenous rehydration is often employed, the emergency department administration of oral ondansetron to children with dehydration and vomiting secondary to gastroenteritis results in significant monetary savings compared to a no-ondansetron policy.

*Please see later in the article for the Editors' Summary*

## Abstract 2

Stephen Freedman and colleagues performed a cost analysis of the routine administration of ondansetron in both the United States and Canada and show that its routine administration to eligible children in such settings could provide substantial benefit.

## Editors' Summary

**Background:**

Although many episodes of gastroenteritis in children are mild and can be managed with oral fluids, including oral rehydration therapy (ORT), some cases are severe enough to require hospital admission for intravenous fluids. Administration of an antiemetic (a drug that reduces nausea and sickness) can be clinically effective, especially ondansetron, (a drug that belongs to a class of drugs known as selective serotonin receptor antagonists), which is safer than other antiemetics, such as promethazine and prochlorperazine, and in which there is good evidence to support its effectiveness in improving the success of ORT in children with gastroenteritis. Furthermore, studies have shown that administration of ondansetron decreases the risk of further vomiting, and hence the need for intravenous rehydration, and immediate hospital admission. However, despite the proven clinical benefits of ondansetron, clinical practice guidelines continue to recommend against the use of antiemetics in gastroenteritis because the evidence of cost savings is not yet clear. Last year, the UK's National Institute for Health and Clinical Excellence recommended that such a cost analysis should be a key research priority in pediatric gastroenteritis.

**Why Was This Study Done?:**

This study—which is an economic analysis—was conducted in response to the various calls for the need to demonstrate the cost effectiveness of ondansetron in the management of pediatric gastroenteritis.

**What Did the Researchers Do and Find?:**

The researchers analysed the costs of the administration of oral ondansetron in both the US and Canada, if routinely given to children with gastroenteritis-induced vomiting and dehydration in the emergency department setting. In addition, the researchers calculated the incremental cost of ondansetron per quality-adjusted life-year (QALY) gained from a health care perspective, compared to a regimen without ondansetron administration. The authors conducted a particular type of statistical analysis, known as decision tree analysis, to compare the two treatment options—administering ondansetron and not administering ondansetron in addition to ORT, with the clinical outcomes (further vomiting, intravenous rehydration, and hospitalization) determined on the basis of the documented efficacy of ondansetron. In addition, the researchers conducted their analyses from both the societal perspective (which included all costs, both direct—the resources required to produce a service; and indirect—productivity costs) and the health care payer's perspective. The US and Canada use similar medical resources, management programs, and treatment guidelines, but as prices differ dramatically (for example, the cost of hospitalization in the US is 8-fold higher than that in Canada), the researchers conducted a separate analysis for each country.

On the basis of data from the US, the researchers found that the administration of ondansetron to eligible children would prevent approximately 29,246 intravenous insertions and 7,220 hospitalizations every year with an annual saving of US$65.6 million to society and US$61.1 million to payers of health care costs if this drug was given routinely. When using Canadian data, the researchers found that the administration of ondansetron to eligible children would prevent 4,065 intravenous insertions and 1,003 hospitalizations every year, with an annual saving of CDN$1.72 million to society and CDN$1.18 million to payers of health care costs if this drug was given routinely.

**What Do These Findings Mean?:**

The results of this study show that the emergency department administration of oral ondansetron to children with dehydration and vomiting secondary to gastroenteritis results in significant monetary savings from both societal and health care perspectives compared to a policy that does not include ondansetron administration. Furthermore, the societal savings are probably an underestimate because in their model, the researchers assumed that only 10% of children with gastroenteritis presenting to an emergency department would meet eligibility criteria (in reality, this proportion would likely be higher). In addition, the researchers did not include estimates for ondansetron administration in the clinic or private office setting, as although such use is common, no estimates of eligibility and efficacy were available.

Therefore, in addition to being clinically beneficial, the administration of oral ondansetron to children with dehydration and vomiting secondary to gastroenteritis is also economically advantageous.

**Additional Information:**

Please access these Web sites via the online version of this summary at http://dx.doi.org/10.1371/journal.pmed.1000350.

- Patient UK and the US National Institutes of Health provide information for patients on ondansetron
- Patient UK provides information on gastroenteritis in children
- BBC Health also provides general information on gastroenteritis
- The Centers for Disease Control and Prevention contains a report on managing acute gastroenteritis among children

## Introduction

The use of antiemetics for children with vomiting is one of the most controversial decisions in the treatment of gastroenteritis in developed countries [1]. Although oral rehydration therapy (ORT) is recommended for children with mild to moderate dehydration, it remains underused [1],[2]. In one survey, over a third of pediatricians indicated that vomiting is a contraindication to ORT [3], while 86% of pediatric emergency medicine physicians who responded to a survey indicated that they are more likely to administer intravenous rehydration when vomiting is the major symptom [4]. Physicians frequently prescribe antiemetic agents [5] because they are interested in increasing the success of ORT and reducing the discomforts of vomiting. However, antiemetic agents commonly used in the 1990s such as promethazine and prochlorperazine [5],[6] are associated with frequent and potentially life-threatening side effects [7], which resulted in a negative perception of all antiemetics and a reduction in their use for children with gastroenteritis.

Ondansetron, a selective serotonin receptor antagonist, has been found to be effective in improving the success of ORT in children with gastroenteritis [8]–[13]. A recent meta-analysis [14], reported a decreased risk of further vomiting (absolute risk reduction [ARR] = 21%), intravenous rehydration (ARR = 20%), and immediate hospital admission (ARR = 7%). An increase in stool output without a concomitant increase in health care utilization was reported in some studies. Despite these benefits, clinical practice guidelines continue to recommend against the use of antiemetics in gastroenteritis [1],[2],[15], stating that evidence of cost savings would further support the argument for ondansetron administration [1],[2],[14]–[17]. In April 2009, the National Institute for Health and Clinical Excellence of the United Kingdom listed a cost analysis as a key research priority in pediatric gastroenteritis [15].

We conducted an economic analysis of the emergency department (ED) administration of oral ondansetron to children with vomiting and dehydration secondary to gastroenteritis. The primary objective was to perform a cost analysis of the routine administration of ondansetron in both the US and Canada; the secondary objective was to assess, from a health care perspective, the incremental cost of ondansetron per quality-adjusted life-year (QALY) gained compared to a regimen without ondansetron administration.

## Methods

### Study Design

A cost analysis was conducted to evaluate the overall costs of routinely administering oral ondansetron to eligible children. In this analysis, all costs are expressed in monetary terms. A cost-utility analysis looking at the cost per QALY gained was additionally conducted. In our model, the strategies compared were administering ondansetron in addition to routine care (ORT) versus ORT alone over a 1-y period for the entire populations of Canada and the US.

**Figure 1:**
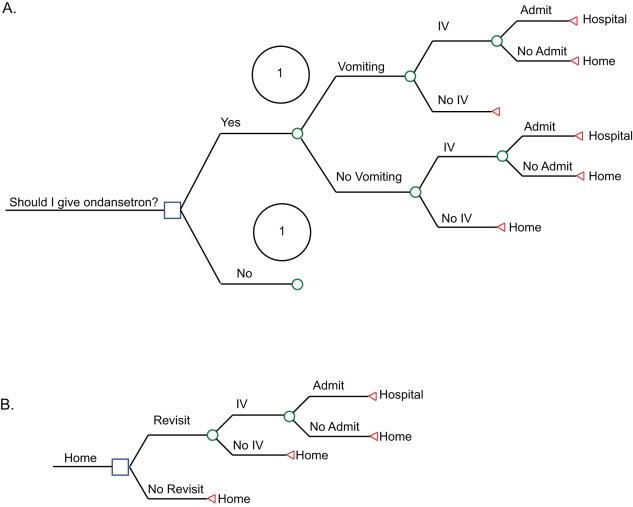
Tree structure used in the decision model. The tree structure was too large to demonstrate using a single figure and has been divided up into two sections labeled (A) and (B). Entry into tree (A) requires that the child meet eligibility criteria (vomited on the day of presentation and evidence of dehydration). The two strategies evaluated are presented after the decision node, indicated by a square in (A). Each possible outcome (i.e., vomiting, intravenous rehydration) is presented on a tree branch after a chance node, shown as a circle on the tree. The complete tree is symmetrical beginning from the left of (A) and moving to the right, through (B) with the “yes” and “no” arms being identical as represented by the number 1. The costs and outcomes were entered at the payoff node, indicated by a triangle at the end of the models in both (A) and (B). An individual simulated patient can be followed through the tree by starting with the treatment choice in (A) and moving through the tree. The patient will have the potential to develop an outcome on the basis of the probabilities present at each chance node and are presented in Table 1. At the end of the tree, the total cost for that simulated patient is calculated on the basis of the treatment received and the outcomes experienced. Yes implies ondansetron is administered to all in addition to ORT; no implies standard of care (ORT) is administered but ondansetron is not administered. IV, intravenous.

A decision tree (Figure 1), was constructed to compare the two treatment options—administering ondansetron and not administering ondansetron in addition to ORT. Under the “yes” and “no” to ondansetron administration arms, vomiting could continue, which could lead to intravenous rehydration and to hospitalization. Children in both arms could be discharged to home and require a repeat ED visit. The proportions experiencing the outcomes are based on the efficacy of ondansetron (Table 1) [8]–[14]. Costs were not discounted as the time horizon is less than 1 y [18].

**Table 1:** Ondansetron efficacy estimates used in the cost analysis.

Variable	Distribution	Proportion Experiencing Event		Estimates in Sensitivity Analysis^a^		Source
		Ondansetron (%)	No Ondansetron (%)	Ondansetron (%)	No Ondansetron (%)	
Vomiting	Normal	15	36	10–19	30–42	[14]
Intravenous rehydration if vomits in ED	Normal	41	47	25–58	35–58	[8],[9]
Intravenous rehydration if no vomit in ED	Normal	4	15	1–8	8–22	Data provided by authors [8],[9]
Admission to hospital if IV fluids administered^b^	Normal	23	30	8–37	20–41	Data provided by authors [8]–[10]
ED revisit	Normal	12	10	8–17	6–15	Data provided by authors [14]
Admission to hospital during revisit	Gamma	44	29	26–65	13–51	Data provided by authors [14]
Vomit initial dose of ondansetron (require readministration)	Normal	5	0	1–9	0	Data provided by authors [8]

a 95% confidence intervals employed from meta-analysis data when available.

b Assumes that all children admitted from the ED received intravenous rehydration in the ED.

The analyses were conducted from both the societal perspective and the health care payer's perspective. The former includes all costs, both direct (the resources required to produce a service) and indirect (productivity costs). Since similar medical resources, management programs, and treatment guidelines are employed in the US and Canada, but prices differ dramatically, separate analyses were performed using data from each country.

### Data Sources and Definition of the Study Population

The data sources included administrative data to derive the number of ED visits and hospitalizations; administrative and micro-costing data to derive costs; and meta-analysis and randomized clinical trial data to determine the probability of events.

For the US model, the number of annual gastroenteritis ED visits was derived from the State Emergency Department Databases (SEDDs) developed as part of the Healthcare Cost and Utilization Project (HCUP) sponsored by the Agency for Healthcare Research and Quality (AHRQ). The weighted national estimate of the total number of admissions recorded in the Kids' Inpatient Database (KID) was used to calculate the number of pediatric ED visits per year. Canadian estimates were derived from the National Ambulatory Care Reporting System (NACRS) available from the Institute of Clinical Evaluative Sciences (ICES). Since estimates for ED visits were only available for the province of Ontario, the national estimate was derived from the number of ED visits multiplied by the proportion of the Ontario to Canadian population less than 15 y old from the 2006 National Census [19]. In both countries, estimates of eligibility for ondansetron treatment were derived using restrictive International Classification of Diseases (ICD) codes (Text S1) and data from the 2005 calendar year [20].

A multinational expert panel concluded that 10%–15% of all children presenting to EDs with acute gastroenteritis would meet eligibility criteria for ondansetron administration as defined in the clinical trials upon which efficacy estimates are based (mild to moderate dehydration and recent vomiting). Given the uncertainty around this estimate, a single centre, 4,000-patient chart review (The Hospital for Sick Children) revealed that 16% of children met eligibility criteria (unpublished data). Our analysis was conducted assuming 10% of all children would be eligible to reflect uncertainty across centres in this regard and ensure that our estimate of benefit, if found, would be conservative.

### Decision Model Parameters

The probabilities of events in the decision model and estimates of ondansetron efficacy were based on the summary findings of a recent meta-analysis [14] of ondansetron use in children with gastroenteritis (Table 1). When the desired probabilities were unavailable from the meta-analysis or previously published randomized clinical trials [8]–[12], the authors of the studies were contacted to provide the raw data (SBF, MJS, DMS) [8],[9],[14]. When estimates or assumptions were required, those that would bias the results against ondansetron use were selected.

### Costs

Medical costs included in the analysis were those related to medication acquisition, dispensing, hospitalization, ED visits, and intravenous rehydration (Table 2). Included in the cost estimates were the costs of supplies, personnel, and nursing and physician services. Nonmedical costs included were those related to foregone earnings of parents, the consumption of special foods and oral rehydration solution, extra diapers, and travel.

**Table 2:** Management costs for intervention and outcome events used in the cost analysis and ranges considered.

Management Cost	US: US$ (range)	Canada: CDN$ (range)
**Medical costs**		
Hospitalization	7,539 (5,654–9,424)^a^	955 (716–1194)
ED visit	704 (528–879)^b^	189 (141–236)
Physician costs, inpatient	273 (205–341)	183 (137–228)
Physician cost, ED	61.31 (45.98–76.64)	40.93 (30.70–51.16)
Intravenous insertion	194 (145–243)	84 (63–106)
Ondansetron	26.57 (19.93–33.21)	12.86 (9.64–16.07)
**Nonmedical costs**		
Forgone earnings of parent/hour	19.29 (14.47–24.11)	18.55 (13.91–23.19)
Special food, ORS	24.00 (18.00–30.00) [37]	27.27 (20.45–34.09)
Extra diapers	9.00 (6.75–11.25) [37]	10.23 (7.67–12.78)
Travel	19.00 (14.25–23.75) [37]	21.59 (16.19–26.98)

Costs are adjusted to 2006 US$ and CDN$ respectively.

a Data obtained from weighted national estimates from HCUP State inpatient Databases (SID) 2005 and the AHRQ, on the basis of data collected by individual states and provided to AHRQ by the states.

b Data obtained from weighted estimate derived from SEDD 2005 and the AHRQ on the basis of data collected by individual states and provided to AHRQ by the states.

ORS, oral rehydration solution.

The mean length of stay for hospitalized children with gastroenteritis reported in KID is 2.1 d and the number of workdays lost by the caregivers of children with gastroenteritis evaluated in outpatient settings is 0.7–2 d [21]–[24]. We estimated that an 8-h work day is lost for all outpatient visits not requiring intravenous rehydration (no IV, home), that 16 h of work are lost for all outpatient visits requiring intravenous rehydration but not admission (IV, no admit, home), and that 24 h of work are lost per child admitted (IV, admit, hospital).

For analysis purposes, the Canadian dollar was valued at US$0.88 (2006) with a sensitivity range of (US$0.80–US$1.00). All cost data reported are adjusted for use of 2006 as the base year. Conversions were conducted using indices commonly employed to adjust for inflation [25], the Bureau of Labor Statistics Consumer Price Index Medical Care inflation rates [26],[27], and the Statistics Canada Health and Personal Care Consumer Price Index [28].

**US:** The point estimate charge for oral ondansetron administration (US$26.57) was the average wholesale price (AWP) [29]. The AWP was selected as it is almost always greater than the wholesale acquisition cost and lists prices that are much higher than public or private payers are likely to pay, reflecting the opportunity cost [30], and has been used previously for similar analyses [31]. ED visit and hospitalization costs were derived from the KID and the SEDD, respectively, from 2006. These data sources reflect the amount the hospital charged for the entire hospital stay (or ED services if using the SEDD), but do not include professional fees. Hospital charges were then converted to costs using the HCUP cost-to-charge ratios on the basis of hospital accounting reports from the Centers for Medicare and Medicaid Services. Intravenous insertion costs and physician fees were derived from the 2008 Physicians' Fee Reference 50th percentile [32]. A proportional adjustment to the mean cost of an ED visit, as provided by SEDD, was performed to account for the estimated (25%) frequency of intravenous rehydration [33]. Nonmedical costs included lost time from work valued at the parent's wage rate, calculated by multiplying the hourly wage by the number of hours of work missed. Caregiver hourly wages were derived from the June 2006 National Compensation Survey [34]. Length of hospitalization was estimated from the 2006 KID and the number of workdays lost by the caregivers of children with gastroenteritis evaluated in outpatient settings was derived from the literature [21]–[24]. Data regarding length of the ED stay was derived from unpublished data collected during an earlier clinical trial [8]. Owing to the transient increase in diarrhea reported amongst children treated with ondansetron [14], diaper costs were estimated to be 33% greater in the ondansetron arm.

**Canada:** The point estimate cost for oral ondansetron (CDN$13.09) was derived from the average of five Canadian provincial drug benefit plans that reimburse for its use. Cost estimates for ED visits, hospitalizations, and intravenous insertions were derived from The Hospital for Sick Children's ED and inpatient average costs for supplies and personnel, and included Ontario Health Insurance Plan physician billing costs for consultation, continued care, ED visits, and nursing time when appropriate. Wages for estimation of productivity costs were derived from the Statistics Canada CANSIM database [35]. All other cost estimates employed the same assumptions as described above for the US.

### Cost Analysis

Both deterministic estimates and probabilistic analyses were undertaken to derive estimates of the value of an all-usage versus a no-usage policy regarding ondansetron administration to eligible children. The Monte Carlo microsimulation model used the decision trees (Figure 1) with the associated probabilities and distributions (Table 1), and costs (Table 2) associated with each outcome. The number of trials used in the Monte Carlo microsimulation model equaled the number of eligible children. The Monte Carlo microsimulation model was created using Tree Age Pro Suite 2009 (Data TreeAge Software Inc., Release 1.0.2, 2009).

One-way sensitivity analyses were conducted to determine how the model's results changed as the key assumptions were varied over plausible ranges (75%–125%). Each one-way sensitivity analysis was presented in a Tornado diagram to evaluate the impact of the maximum and minimum expected values of all probabilities and costs. Variables are ordered with those with the broadest range of impact on the top; progressively narrower ranges of impact are placed below, giving an appearance similar to that of a tornado. The maximum variation in the variable deemed to be most important in the tornado analysis was then employed for sensitivity analysis purposes.

### Cost-Utility Analysis

The cost-utility analysis employed previously published QALY estimates, which were derived on a cohort of 450 caregivers from the US [36]. The available QALYs are for moderate (0.93) and severe gastroenteritis (0.90), and these states closely resemble the “no IV, home” arm (moderate gastroenteritis) and the “IV, admit, hospital” arm (severe gastroenteritis) (Figure 1). To avoid a bias in favour of ondansetron the “IV, no admit, home” arm was assigned values consistent with moderate gastroenteritis.

## Results

### Study Population

The proportion of gastroenteritis ED visits that result in hospitalization in the US is 4.2%. Applying that proportion to the weighted national estimate of the total number of admissions yields a total number of 1,725,493 pediatric ED visits annually. The number of Ontario ED visits was 95,017, and the proportion of the Ontario to Canadian population less than 15 y old is 39.6%. Thus, the annual number of Canadian ED visits is estimated to be 239,813. Employing our eligibility estimate (10%), the numbers of children who are suitable for ondansetron administration are 172,549 in the US and 23,981 in Canada.

### Cost Analysis

**US:** The administration of ondansetron to 10% of all children with ED visits for acute gastroenteritis would prevent 29,246 (sensitivity analysis range, 22,122–32,556) intravenous insertions and 7,220 (3,671–10,990) hospitalizations annually. The deterministic model shows that total costs incurred from the health care perspective in an all-ondansetron use pattern would be US$1,249 per patient (US$935–US$1,561), while a no-ondansetron use pattern, would cost US$1,602 (US$1,202–US$2,002) per patient, providing a net savings of US$353 (US$267–US$441) per patient (Table 3). From a societal perspective, the total costs incurred from an all-ondansetron use pattern would be US$1,500 per patient (US$1,119–US$1,867), while a no-ondansetron use pattern would cost US$1,879 (US$1,403–US$2,336), providing a net savings of US$379 (US$284–US$469) per patient.

**Table 3:** Cost analysis of ondansetron administration to children with vomiting and dehydration secondary to acute gastroenteritis who are treated in an ED.

Economic Analysis Model and Perspective	US: US$^a^	Canada: CDN$^a^
**Health Care Perspective-Deterministic Model**		
Net savings per patient	353 (267–441)	49 (37–59)
Net savings total population	60.9 million (46.1–76.1)	1.18 million (0.89–1.41)
**Health Care Perspective-Monte Carlo Model**		
Net savings per patient	355 (270–439)	48 (36–60)
Net savings total population	61.4 million (46.6–75.7)	1.15 million (0.86–1.44)
**Societal Perspective-Deterministic Model**		
Net savings per patient	379 (284–469)	72 (48–79)
Net savings total population	65.4 million (49.0–80.9)	1.72 million (1.15–1.89)
**Societal Perspective-Monte Carlo Model**		
Net savings per patient	380 (283–467)	71 (47–76)
Net savings total population	65.6 million (48.8–80.6)	1.70 million (1.13–1.82)

a Sensitivity analyses in parentheses. Costs are adjusted to 2006 US$ and CDN$ respectively.

The probabilistic model revealed that for each child treated, the total costs from an all-ondansetron use pattern was US$1,251 (US$935–US$1,564) while a no-ondansetron use pattern was US$1,606 (US$1,205–US$2,003), saving the health care system US$355 (US$270–US$439). From a societal perspective, the health care system would cost US$1,500 (US$1,119–US$1,867) from an all-ondansetron use pattern, while a no-ondansetron use pattern would cost US$1,880 (US$1,402–US$2,334), leading to a total saving of US$380 (US$283–US$467) for an all-ondansetron use pattern.

The break-even total cost per dose of ondansetron administered (where net savings are as likely as net costs) is US$362 (US$275–US$450) from the health care payer and US$387 (US$293–US$479) from the societal perspectives. At the current mean average wholesale price [32] of US$26.57 per dose, its administration to eligible children would result in a net savings from the health care perspective of US$60.9 (US$46.1–US$76.1) million under the deterministic and US$61.4 (US$46.6–US$75.7) million under the probabilistic models for the entire US population (Table 3). From a societal perspective, the savings would be US$65.4 (US$49.0–US$80.9) and US$65.6 (US$48.8–US$80.6) million under the deterministic and probabilistic models respectively.

**Canada:** Ondansetron administration to all eligible children would prevent 4,065 (3,075–4,525) intravenous insertions and 1,003 (510–1,528) hospitalizations annually. The deterministic model shows that total costs incurred from the health care perspective in an all-ondansetron use pattern would be CDN$331 (CDN$247–CDN$416) per patient, while a no-ondansetron use pattern, would cost CDN$380 (CDN$284–CDN$475) per patient, resulting in a net savings of CDN$49 (CDN$37–CDN$59) per patient (Table 3). From a societal perspective, an all-ondansetron use pattern would cost CDN$582 (CDN$395–CDN$662), while a no-ondansetron use pattern would cost CDN$654 (CDN$443–CDN$741) resulting in a net savings of CDN$72 (CDN$48–CDN$79) per patient.

The probabilistic model revealed that an all-ondansetron use pattern would cost CDN$330 (CDN$248–CDN$415), while a no-ondansetron use pattern would cost CDN$378 (CDN$284–CDN$475), saving the health care system CDN$48 (CDN$36–CDN$60). The societal perspective of the probabilistic model shows an all-ondansetron use pattern would cost CDN$583 (CDN$396–CDN$663), and a no-ondansetron use pattern would cost CDN$654 (CDN$443–CDN$739), resulting in a savings of CDN$71 (CDN$47–CDN$76).

From the health care perspective, ondansetron administration would result in savings if it costs CDN$59 (CDN$49–CDN$72) or less per dose. From the societal perspective, the break-even total cost per dose administered is CDN$82 (CDN$60–CDN$91). At the current mean provincial drug benefit plan reimbursement rate of CDN$13.09, its administration to eligible children would result in a net savings from the health care perspective of CDN$1.18 (CDN$0.89–CDN$1.41) million under the deterministic and CDN$1.15 (CDN$0.86–CDN$1.44) million under the probabilistic models for the entire Canadian population (Table 3). From a societal perspective, the savings using a deterministic model would be CDN$1.72 (CDN$1.15–CDN$1.89) million and CDN$1.70 (CDN$1.13–CDN$1.82) million under the probabilistic models, respectively.

### Sensitivity Analysis

**Figure 2:**
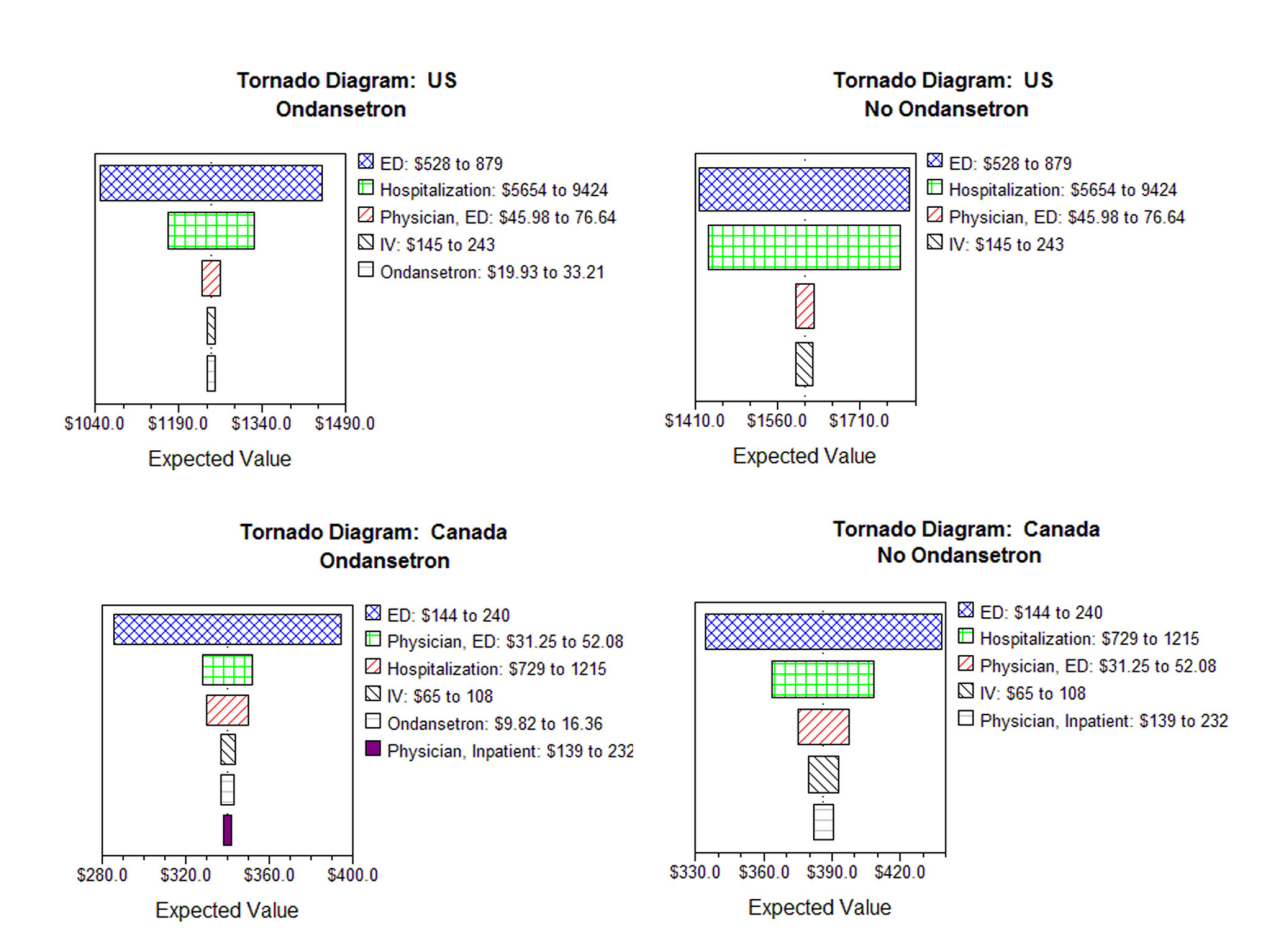
Tornado diagram showing the influence of changing values of any variable on per patient costs. Tornado diagram showing the influence of changing values of any variable on per patient costs when other variables remain at their base values. In the graph, variables are ranked on the basis of their influence (the most influential variable is on the top). Only variables that had more than 1% effect in the expected value were included. IV, intravenous.

When varying individual variables (Table 4), the savings range from US$151–US$576 in the US and CDN$20–CDN$82 in Canada. In both countries, the tornado diagrams (Figure 2) reveal that the cost of the ED visit had the greatest impact on both the ondansetron and no-ondansetron pathways. Varying the cost of physician inpatient fees had the smallest impact in the models.

**Table 4:** Range of costs associated with maximal variation in individual parameters included in model.

Focus of Sensitivity Analysis	US: US$ Range of Total Savings/Patient Administered Ondansetron—Health Care Perspective^a^	Canada: CDN$ Range of Total Savings/Patient Administered Ondansetron—Health Care Perspective^a^
**Costs**		
Hospitalization	265–443	37–60
Physician cost, inpatient	350–356	46–51
Physician cost, ED	353–354	48–49
ED visit	349–358	48–50
IV insertion	345–361	46–53
Ondansetron	346–360	45–52
**Proportions: Ondansetron administered**		
Vomiting	325–389	44–56
IV insertion if vomits	271–432	36–63
IV insertion if does not vomit	288–402	39–59
Admission if IV fluids administered	252–461	35–65
ED revisits	243–441	27–69
ED revisit resulting in admission	274–421	38–59
Vomiting initial dose of ondansetron	352–354	49–50
**Proportions: Ondansetron not administered**		
Vomiting	306–401	41–57
IV insertion if vomits	246–452	31–66
IV insertion if does not vomit	242–465	30–68
Admission if IV fluids administered	151–576	20–82
ED revisits	288–435	34–68
ED revisit resulting in admission	306–418	42–59

a Due to nearly identical data for health care and societal perspectives, we have displayed only health care perspective to facilitate clarity. Costs are adjusted to 2006 US$ and CDN$ respectively.

IV, intravenous.

### Cost-Utility Analysis

For every additional ondansetron tablet used, 0.0015 (0.00085–0.0022) QALYs would be gained. In the US, an all-ondansetron administration policy would result in 160,677 (160,308–160,850) QALYs per year, while a no-ondansetron usage policy would result in 160,411 (159,930–160,703) QALYs per year, resulting in a net gain of 266 (146–377) QALYs per year. In Canada, an all-ondansetron usage pattern would result in 22,198 (22,147–22,221) QALYs per year, while a no-ondansetron usage pattern would result in 22,161 (22,094–22,201) QALYs per year, resulting in a net gain of 37 (20–52) QALYs per year. Since the program results in a net cost reduction, we did not evaluate the cost per QALY gained.

## Discussion

We estimate that every year, oral ondansetron administration to eligible children in the ED would prevent intravenous insertion in approximately 30,000 children in the US and 4,000 in Canada. Over 8,000 admissions per year would be avoided in these countries combined. Ondansetron administration to the appropriate group of children would additionally result in an incremental savings from the societal perspective of US$66 million per year in the US and CDN$1.7 million in Canada. Thus, it is clear that ondansetron administration to children with vomiting and dehydration in the ED is a dominant strategy (i.e. ondansetron administration results in improved outcomes and reduced costs).

Several systematic reviews have evaluated the evidence of benefit derived from the use of ondansetron, and while they all had similar findings, their endorsement of ondansetron use was made in conjunction with the need for an economic analysis. The 2009 Cochrane Review included four clinical trials, did not include a meta-analysis, and had a primary objective that was not dealt with by any of the studies (time to achieve cessation of vomiting) [17]. The review concluded that while ondansetron may reduce the amount of vomiting, and the numbers of children requiring intravenous rehydration and hospitalization, it stressed the need to conduct a cost analysis [17]. A meta-analysis published in 2007, which also included four trials, concluded that ondansetron use is associated with some clinical benefit [16]. However, the authors state that there is insufficient evidence to recommend the routine use of ondansetron and that future studies need to address the economic implications of using ondansetron. A more recent systematic review and meta-analysis, which included six studies, concluded that the symptomatic relief and avoidance of invasive therapies seen with ondansetron use suggest that it is beneficial when administered to moderately ill children with gastroenteritis. This meta-analysis included a study that was excluded from the Cochrane Review because of the inclusion of participants up to the age of 22 y [12], and a study that appears to have not been retrieved by their search strategy [11]. It additionally included two studies [10],[11] not included in the earlier meta-analysis [16]. Thus, the meta-analysis [14] data upon which our effectiveness estimates are based are the most complete in the literature and more definitively recommend the ED use of ondansetron. Nonetheless, even the authors of the meta-analysis with the strongest conclusions state that formal cost analyses should be performed [14]. Thus, our data answer the important questions raised in these recent reviews and can be employed to aid clinicians and health care administrators when making decisions at patient and societal levels.

We can compare the economic implications of our findings to the benefits incurred from other therapies employed in the treatment of pediatric gastroenteritis. For example, the now endorsed US rotavirus vaccination program requires the administration of three vaccine doses to 4 million infants to result in 44,000 fewer hospitalizations per year [37],[38]. The administration of ondansetron to 172,549 children in the US (4% as many as the vaccine program) would result in approximately 8,000 fewer admissions (18% as many as the vaccine program). In addition, the rotavirus vaccination program would cost an extra US$515 million to the health care system, while routine ondansetron administration would save the health care system US$61 million. On a patient outcome level, the benefits of ondansetron administration can be compared with those seen with the use of zinc, which is endorsed by the World Health Organization and United Nations Children's Fund [39]. Using the Lives Saved Tool methodology, zinc administration to children with diarrhea in developing countries is estimated to decrease the relative risk of hospitalizations by 23% (95% confidence interval [CI] 15%–31%) [40], while the benefit seen with ondansetron administration in developed countries is a 48% relative risk reduction (95% CI 18%–67%). As ondansetron use is a dominant strategy, being both clinically and economically beneficial, its use, in the appropriate clinical situation should be encouraged.

The National Institute for Health and Clinical Excellence conducted a probabilistic sensitivity analysis and found that the results were not sensitive to parameter uncertainty with ondansetron being the dominant strategy in 99.96% of the simulations [15]. However, their model did not incorporate the cost of increased diarrheal events, repeat medication administration due to vomiting, and the need for future hospital visits and the outcomes at those encounters. Despite incorporating these elements into our model, and conducting our analysis employing cost estimates from two different countries, our findings were similar. We did, however, detect a significant difference in break-even prices between countries. This difference is primarily driven by the large role played by the cost of hospitalization in the US, which is approximately 8-fold that in Canada, and the 4-fold difference in ED visits.

Our study has several limitations in the estimates of disease burden and costs. While the numbers of potentially eligible patients were derived from large databases, our primary analysis assumed that only 10% of children with gastroenteritis who present to an ED would meet eligibility criteria. This number is likely an underestimation based on analysis of all cases of gastroenteritis at a Canadian institution where 16% were found to be eligible and a report from two EDs in the US where ondansetron was administered to 58% of more than 34,000 children with gastroenteritis [41]. We additionally did not include estimates on the use of ondansetron in the clinic or private office setting as estimates of eligibility and efficacy are not available, though such use is becoming very common [42]. Thus, our conclusions likely underestimate the total societal savings from the appropriate use of ondansetron.

This analysis focused on a specific population of children with vomiting and clinical evidence of dehydration evaluated in an ED setting. The results should not be extrapolated to children without evidence of dehydration as these children are less likely to experience the costly outcomes of intravenous rehydration and hospitalization. Additionally, no clinical data exist to support the administration of multiple doses of ondansetron or its use in the clinic or private office settings. While accepted as a very safe class of drugs, serotonin receptor antagonists may cause minor side effects such as constipation, diarrhea, headaches, and light-headedness [43]; rarely, they may be associated with more severe reactions [44],[45].

While it is clear that vomiting is a frequent cause of ORT failure in developed countries leading to the use of intravenous rehydration and hospitalization, clinicians working in developing nations do not find vomiting to be as significant a barrier to the success of ORT. In fact, the overall acceptance of ORT in countries such as the US, has lagged far behind that in many developing countries [46]. Although meta-analyses of clinical trials conducted in developed countries have documented only a 5% ORT failure rate, this is much lower than what happens outside of oral rehydration clinical trials [47]. This finding is evidenced by the 34% failure rate in the placebo groups in the ondansetron clinical trials [14], despite claiming that appropriate ORT protocols were followed. A multitude of explanations are possible for this discrepancy; however, a key variable is the preference of parents in the US for the administration of intravenous rehydration [48]. However, of those parents who selected intravenous rehydration, 53% stated that they would choose oral rehydration if an oral medication were available that would significantly decrease vomiting. Thus, it seems that ondansetron may play a key role in promoting the use of ORT in environments where the overall usage of ORT is suboptimal. Hence, at present, usage of ondansetron in children with gastroenteritis should be limited in the developing world where the emphasis should remain on ORT alone.

The strengths of this study include the use of original data, which allowed us to estimate the frequency of outcomes not previously reported. Despite using wide margins of error in the sensitivity analysis, the conclusions did not change significantly. Lastly, our conclusions are strengthened because the analysis included cost estimates from two countries, with different health care systems. Despite this important economic distinction, our analysis concluded that ondansetron administration would result in cost savings in both countries.

In addition to being clinically beneficial, the administration of oral ondansetron to children with dehydration and vomiting secondary to infectious gastroenteritis is economically advantageous, making it a dominant treatment strategy. On the basis of the available clinical data and our cost analysis, the use of ondansetron should become routine in North American EDs in order to reduce both the burden of disease on children and the costs to society and health care systems.

## Supporting Information

Text S1 International Classification of Diseases (ICD) coding employed to derive estimates of eligibility for ondansetron treatment. ICD-9 CM coding was employed in the US, while ICD-10 coding was employed to derive Canadian estimates.
(0.02 MB DOC)

## Abbreviations

AHRQ: Agency for Healthcare Research and Quality
ED: emergency department
HCUP: Healthcare Cost and Utilization Project
KID: Kids' Inpatient Database
ORT: oral rehydration therapy
QALY: quality-adjusted life-years
SEDD: State Emergency Department Databases
