# Supplementary material for: Oral Ondansetron Administration in Emergency Departments to Children with Gastroenteritis: An Economic Analysis
Source: PLoS Med. 2010 Oct 12;7(10):e1000350. doi: 10.1371/journal.pmed.1000350 (PMC2953527; doi:10.1371/journal.pmed.1000350)
Supplement: Text S1 — International Classification of Diseases (ICD) coding employed to derive estimates of eligibility for ondansetron treatment. ICD-9 CM coding was employed in the US, while ICD-10 coding was employed to derive Canadian estimates. (0.02 MB DOC) [file pmed.1000350.s001.doc]

**Appendix I**

We included the following International Classification of Diseases, 9th Revision, Clinical Modification (ICD-9 CM) codes in the United States:

- diarrhea of determined etiology (viral [008.6 and 008.8])
- diarrhea of undetermined etiology, including infectious cases (009.0-009.3)
- dehydration (276.51)
- nausea with vomiting (787.01)
- vomiting alone (787.03)
- diarrhea not otherwise specified (787.91)

Canadian estimates were derived by converting ICD-9 CM to the following International Classification of Diseases, 10th Revision (ICD-10) codes:

- intestinal infectious disease (A00-A09)
- volume depletion (E86)
- nausea and vomiting (R11)
- non-infectious gastroenteritis (K52.9)
